# Body image perception and body composition in early adolescents: a longitudinal study of an Italian cohort

**DOI:** 10.1186/s12889-021-11458-5

**Published:** 2021-07-12

**Authors:** Stefania Toselli, Alessia Grigoletto, Luciana Zaccagni, Natascia Rinaldo, Georgian Badicu, Wilhelm Robert Grosz, Francesco Campa

**Affiliations:** 1 Department of Biomedical and Neuromotor Sciences, University of Bologna, 40126 Bologna, Italy; 2 Department of Neuroscience and Rehabilitation, Faculty of Medicine, Pharmacy and Prevention, University of Ferrara, 44121 Ferrara, Italy; 3 Department of Physical Education and Special Motricity, University Transilvania of Brasov, 500068 Brasov, Romania; 4 Department for Life Quality Studies, University of Bologna, 47921 Rimini, Italy

**Keywords:** Body size, Body shape, Health, Fat mass, Adolescence

## Abstract

**Background:**

Adolescence is a sensitive period of life in which everyone faces physical and psychological changes. No longitudinal studies considering changes in body image perception and body composition in Italian adolescents have been carried out. The aims of this study were to evaluate the longitudinal change in body composition and weight status in a sample of Italian students of both sexes over the 3 years of middle school and to analyse the influence of these parameters on the perception and satisfaction of one’s own body image.

**Methods:**

Sixty-four males and seventy females were followed longitudinally from 11 to 14. Age at first measurement was 11.8 ± 0.3 yrs. in males and 11.9 ± 0.3 yrs. in females, then the students were measured again after 1 year and 2 years. Anthropometric measurements were collected and body composition was assessed by skinfolds. Maturity status was detected by age at menarche in females and by estimated age at peak height velocity in males; sports practice was assessed by a questionnaire. Body Silhouette Charts were used to assess body image perception. The degree of body image dissatisfaction and improper perception of weight status were evaluated. Univariate and multivariate analyses were applied.

**Results:**

Height, sitting height, leg length, and weight increments were higher in males than in females, and in both sexes the sitting height increment was higher than that of leg length. Skinfold thicknesses and percentage of body fat, showed a decrease in males and an increase in females over the 3 years. About 90% of the sample practiced sport during the 3 years. No significant variations in body image perception were observed among repeated measures but significantly differences were observed between sexes. Although the girls showed a lower incidence of overweight and obesity than boys, girls had a higher dissatisfaction than males. Males were less accurate in one’s perception of one’s own weight status.

**Conclusions:**

The changes in body composition observed in the sample of the present study were in accordance with their maturity stage. An increase in parameters connected with adiposity is observed in females and a decrease in males. Body image perception did not seem to change with growth, but associations were found between body image perception and BMI and sex. Monitoring body image perception in young adolescents, especially in females and in overweight/obese subjects, is a priority to prevent nutritional disorders.

## Background

Early adolescence is generally considered the period between 10 and 14 years [1] and starting from this period, during the middle school, most students face physical and psychological changes. This early stage of adolescence is characterised by growth spurt and is soon followed by the development of secondary sexual characteristics. Given the changes that characterize adolescent development, it is not surprising that there are also significant changes in the types and frequency of health problems and psychological disorders during this period, as compared to childhood [2]. Many of the health-related behaviours that arise during adolescence have implications for both present and future health and development. For example, obesity in early adolescence not only compromises adolescent development but it also predicts health-compromising obesity in later life, with serious implications for public health [3].

Adolescence is in fact considered a critical window for the development of overweight and obesity and there is consistent evidence showing that excess weight in youth tends to track into adulthood [4, 5]. The evaluation of these disorders in adolescents is thus a priority. Overweight and obesity are public health problems and the prevalence among 11-year-olds was greater than 10% for boys in four countries in Europe (Croatia, Greece, Portugal and the Former Yugoslav Republic of Macedonia), but only two countries (Greece and Italy) had a prevalence of more than 5% of overweight and obese girls. Obesity during adolescence is associated with morbidities during this phase and also throughout adult life, with eating disorders (ED) being the third most common chronic disease in adolescence, behind only obesity and asthma [6, 7]. Nevertheless, longitudinal data regarding overweight/obesity prevalence during early adolescence in Italy are lacking.

The fast morphological and psychosocial changes occurring during this period greatly influence body perception [8, 9]. Adolescence represents a critical stage in the development of positive or negative body image [10, 11]. Rapid changes during adolescence in shape and weight due to puberty interact with socio-cultural contexts in influencing body image perceptions [10]. Weight misperception, a perceptual aspect of body image relating to over- or under-estimation of weight, is a separate construct from body dissatisfaction [12–17], since one can be quite accurate in the perception of one’s own size and shape, and yet still be dissatisfied. The nurturing of a healthy body image perception is a challenge during adolescence. Early adolescence represents a key developmental transition. The examination of body image during this transitional period is imperative, since distinct and significant changes occur at these times. During early adolescence, for example, most girls place high importance on peer acceptance [18]. Among adolescents, there is often a desire and constant search for physical characteristics other than reality [19], which can cause body image dissatisfaction [9, 20, 21]. In today’s societies, there is an idealisation of a perfect body, which, in adolescents, if not achieved, could lead to body image disorders, in addition to the effects on health and behaviour [22–24].

There are several biological factors that influence the risk for body image disturbance during adolescence: age, sex, puberty, and body composition [25], and everyone deserves attention. Body mass index is the most commonly used anthropometric indicator in the body image literature. However, BMI has been criticized because it does not discriminate among the various components of body composition, namely fat mass (FM) and fat-free mass (FFM, including muscle mass, bone, and vital organs); BMI can be considered a raw body composition’s indicator [26]. Nevertheless, the assessment of body composition has been rarely considered in body image literature and this has led to an incomplete understanding of how the influence of BMI on body image may differ from that of FM, FFM and of parameters connected with body fat (skinfolds, waist and hip circumferences and waist-hip and waist-height ratios) [26–28]. This aspect is not to be underestimated, especially considering the sex differences in body composition and in body image perception and dissatisfaction. As regards puberty, research has particularly focused on the association between the onset and progression of sexual maturation and psychological and behavioural health consequences [29]. The continuous changes in body growth and psychological development can determine different levels of dissatisfaction of the body image between the different stages of pubertal development [30]. Puberty or changes in maturity status are the most salient developmental milestones during early adolescence and have important implications for self-esteem, body image and psychosocial adjustments [31]. For example, physical self-perception may become less positive with advancing maturity status possibly because of increased adiposity and body dissatisfaction [32, 33]. Also for this aspect, the studies related to the perception of the body image are limited [30, 31, 34].

Nutritional issues in adolescence are mainly characterized by increased energy and nutrient requirements and changes in dietary habits, which could induce overweight and obesity [35]. Adolescents frequently follow eating and behavioural patterns characterised by high-energy (mainly fat) food, skipping daily meals, low fruit and vegetables consumption and an increasingly sedentary routine with many hours in front of the computer and television screen and fewer hours of physical exercise [35]. These kinds of behaviour could determine a greater effort to maintain a lean body and achieve the ideal standard of beauty imposed by the media and society [36].

Change in body composition and weight status during this period can play a determinant role in future outcomes and have socio psychological consequences. The main challenge in clarifying these issues is the lack of longitudinal repeated measures needed to assess developmental changes. Thus, the aim of this study was to examine the trends in body composition and in the prevalence of overweight and obesity, assessed using the Cole cut-off values by sex and age [37, 38], in Italian adolescents followed longitudinally over the 3 years of middle school, also taking into account their maturity status. In addition, we sought to determine whether body composition and weight status during adolescence were associated with the risk of developing body image disturbance. In our opinion, a comprehensive body composition measure, rather than BMI alone, may be needed to disentangle the influences of various body components on body image perception and satisfaction. We expect that adolescence leads to an increase in body image misperception and to a greater dissatisfaction, especially in females, in overweight/obese subjects and in those with higher levels of body fat.

## Methods

### Participants and design

**Fig. 1:**
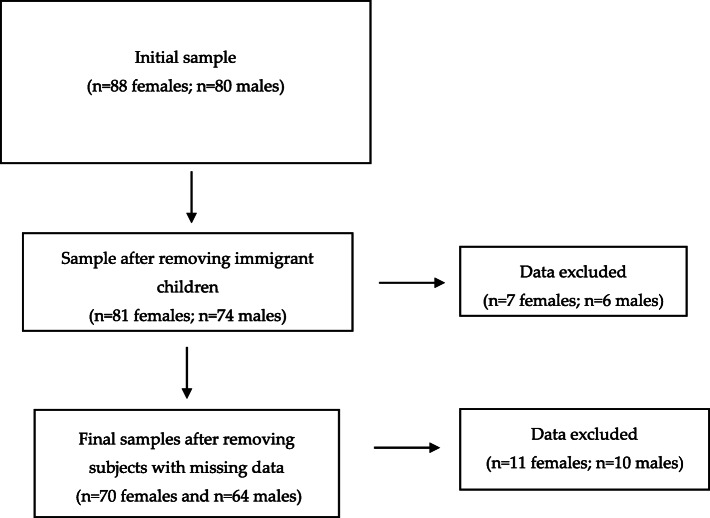
Flow chart illustrating the stages of selection of the final sample

A longitudinal study design was chosen, and data were collected and analysed in a sample of 134 children (64 males and 70 females) attending middle school in the Emilia Romagna region (northern Italy). The students were measured three times at regular intervals: at first measurement mean age was 11.8 ± 0.3 yrs. in males and 11.9 ± 0.3 yrs. in females, then they were measured after 1 year and after 2 years. Only children who received parental written consent and agreed to participate were allowed to take part in the study, without any compensation. The study was approved by the Bioethics Committee of the University of Bologna (approval n. 2.18). A flow chart regarding the selection of the sample is shown in Fig. 1. Only native Italian adolescents with no missing data in all the three measurements were included in the study. Immigrant subjects were excluded since they were not a uniform group, but belonged to different ethnic groups. A trained operator directly collected the anthropometric measurements according to standardized rules described in the Procedures section.

General information about demographic (e.g. sex and age) and sport participation (sport practice - considered as club sports, namely planned sports activities led by staff) and the amount of time involved (hours per week) were collected by a questionnaire. The sport practice frequency of each subject was determined by the hours of sport training during a typical week as declared by the subject.

### Procedures

#### Anthropometry

Anthropometric characteristics (height, weight, lengths, waist and hip circumferences and skinfold thicknesses) were collected in the morning according to standardised procedures [39, 40]. Height and sitting height were measured to the nearest 0.1 cm using a stadiometer (GPM, Zurich, Switzerland), and leg length was derived by the subtraction of sitting height from height. Body weight was measured to the nearest 0.1 kg (light indoor clothing, without shoes) using a calibrated electronic scale. Circumferences were measured to the nearest 0.1 cm with a non-stretchable tape. Skinfold thicknesses were measured to the nearest 0.1 mm using a Lange skinfold caliper (Beta Technology Inc., Houston, TX, USA).

Body mass index (BMI) was calculated as weight (in kilograms) divided by the square of height (in metres). This index was used to assess the weight status of each participant: each subject was thus classified into underweight, normal weight, overweight or obese according to the Cole cut-off values by sex and age [37, 38].

The cormic index was calculated as sitting height (in centimetres)*100 divided by height (in centimetres).

Waist/Hip ratio (WHR) and Waist/height ratio (WHtR) were calculated, and for the last index children were stratified into two categories (≤0.5 and > 0.5); the value of 0.5 was chosen as cut-off for cardiovascular risk [41–43].

Body composition parameters (percentage of fat mass (%F), Fat mass (FM) and Fat free mass (FFM) were calculated using the skinfold equations developed by Slaughter and colleagues [44].

#### Maturity status

In girls, maturity status was detected by asking the girls if they have already reached menarche, and if the answer was affirmative, the date was recorded. In boys, an estimation of the years from peak height velocity (PHV), which is an indicator for the adolescent growth spurt, was made using the equation for boys developed by Mirwald and colleagues [45], which is able to predict maturity offset in youngs [46, 47]. In accordance with Mirwald et al. [45] boys were classified as early, average and late maturers.

Maturity offset = − 9.236 + 0.0002708 (leg length ∗ sitting height) − 0.001663 (age ∗ leg length) + 0.007216 (age ∗ sitting height) + 0.02292 (weight/height).

Since maturity offset represents the time before or after PHV, the years from PHV were calculated by subtracting age at PHV from chronological age.

#### Body image perception

Body image perception was assessed using the Body Silhouette Chart [48]. The students were shown nine male or female silhouettes, ordered in morphology from emaciation to obesity. Subjects were asked to select the silhouette which they believed was most similar to their own (perceived current figure) as well as the silhouette which they most desired (ideal figure). Using the classification reported by Sánchez-Villegas et al. [48], the silhouette series were divided into four categories (underweight, normal weight, overweight and obese). The discrepancy between the perceived current figure and the ideal figure represents the degree of body image dissatisfaction (FID or Feel minus Ideal Discrepancy) [49]. The FID index was calculated by subtracting the score of the figure selected by children as the ideal figure from the one selected as their perceived current Fig. A positive FID score indicates that the perceived current figure was bigger than the ideal figure and a negative score indicates that the perceived current figure was thinner than the ideal Fig. A FID score of 0 indicates no discrepancy (same figure chosen as perceived current figure and as ideal).

Improper perception of weight status was evaluated by means of FAI (Feel weight status minus Actual weight status Inconsistency) [50]: we calculated the FAI inconsistency by subtracting the conventional code assigned to the real weight status of the participant, according to the BMI assessed by Cole cut-off values by sex and age [37, 38], (1 = underweight; 2 = normal weight; 3 = overweight; 4 = obese), from the code of her/his perceived weight status (1 = underweight; 2 = normal weight; 3 = overweight; 4 = obese), obtained using the classification recommended by Sánchez-Villegas et al. [48], which assigns a specific weight status category to each silhouette. According to this classification, silhouettes 1, 2 and 3 correspond to underweight (=1), silhouettes 4 and 5 correspond to normal weight (=2), silhouettes 6 and 7 correspond to overweight (=3), silhouettes 8 and 9 correspond to obesity (=4). A FAI score of zero indicates no inconsistency in weight status perception; a positive score indicates that weight status is overestimated, and a negative score indicates that weight status is underestimated.

### Statistical analysis

The data analysis was performed using Statistica for Windows, version 8.0 (Stat Soft Italia SRL, Vigonza, Padua, Italy).

The suitability of the sample size for each analysis was assessed using the G-Power software 3.1.9.2. An a priori power analysis was conducted to ensure that the number of participants was representative for the purposes of this study. To identify the sample size for the study, we assessed an ‘a priori: computer required sample size- given α, power and Effect Size’ by G*Power (3.1.9.2, Universität Kiel, Germany). A repeated measures ANOVA was selected as the F test of all the test family, imputing the following parameters: α = 0.05; (1 − β) = 0.95; Effect Size f = 0.25; number of groups: 2; number of measurements: 3. The outcomes parameters thus calculated detected a sample size of 44 participants, but additional subjects were involved to ensure availability of data in case of problems with data collection.

Variables normality was verified with the Shapiro–Wilk test. The means and SD of the baseline data (1^st^class) and in the following years (2nd and 3rd classes) were calculated. Percentage frequency was determined for qualitative variables (weight status).

Repeated measures ANOVA was used to assess differences between the values carried out on subjects in each of the 3 years of measurement (from first class to third class), while non parametric ANOVA was used to evaluate the differences among body image parameters, since they are not normally distributed.

Differences in the frequencies were tested by the chi-squared test. Since in the present study the comparison between the weight status consisted of multiple pairwise comparisons (three groups), according to Bonferroni correction, the results were considered statistically significant if p was < 0.05/3 or 0.017.

Backward multiple regression analysis was carried out to assess possible predictors of FID and FAI. Anthropometric variables, sex, weight status and maturity status were included into the model. Predictors input into the model were those found to have significant associations with FID and FAI (i.e., *p* < 0.05), while those with *p* > 0.05 were removed from the model.

## Results

### Anthropometry

Table 1 shows the anthropometric and body composition characteristics of the sample during the three consecutive years in both sexes. Considering sex differences, ANOVA showed significant differences in body proportion (leg length and cormic index), in waist and hip circumferences and relative indexes (WHR and WHtR), in three skinfold thicknesses (suprailiac, medial calf and thigh) and in the sum of skinfolds.

**Table 1:** Descriptive statistics of the sample by sex and repeated measures by ANOVA

	Males						Females						ANOVA					
	I measurement		II measurement		III measurement		I measurement		II measurement		III measurement		Sex difference		Meas. difference		Sex*Meas.	
Variables	Mean	SD	Mean	SD	Mean	SD	Mean	SD	Mean	SD	Mean	SD	F	p	F	p	F	p
height (cm)	151.03	7.11	157.67	8.00	164.30	8.90	153.11	6.31	157.92	5.94	160.55	5.52	0.26	0.608	805.76	0.000	70.24	0.000
sitting height (cm)	77.36	3.74	80.15	4.63	83.49	5.19	79.14	3.80	82.03	3.04	83.59	2.68	3.56	0.061	294.85	0.000	10.92	0.000
leg length (cm)	73.66	4.48	77.52	4.42	80.81	4.74	73.98	3.84	75.89	3.77	76.96	3.83	6.71	0.011	396.24	0.000	70.01	0.000
weight (kg)	44.13	8.07	49.34	8.62	55.06	9.50	44.30	6.98	48.87	6.66	53.36	6.76	0.48	0.491	578.92	0.000	7.20	0.001
Cormic index	51.24	1.40	50.83	1.33	50.81	1.33	51.69	1.41	51.96	1.10	52.08	1.17	24.57	0.000	0.24	0.786	10.01	0.000
BMI (kg/m^2^)	19.53	2.89	19.75	2.61	20.29	2.52	18.85	2.44	19.59	2.46	20.75	2.65	0.18	0.670	58.13	0.000	9.15	0.000
waist circumference (cm)	67.10	6.62	69.53	6.50	71.62	7.13	64.31	5.64	66.05	5.28	67.88	5.93	12.48	0.001	56.55	0.000	1.04	0.353
hip circumference (cm)	81.40	7.20	84.19	6.70	86.99	6.91	84.19	6.40	88.10	6.31	91.88	5.48	11.58	0.001	153.30	0.000	3.04	0.049
WHR	0.71	0.10	0.65	0.05	0.65	0.05	0.72	0.05	0.70	0.04	0.70	0.04	23.75	0.000	25.72	0.000	7.58	0.001
WhtR	0.44	0.04	0.44	0.04	0.44	0.04	0.42	0.04	0.42	0.04	0.42	0.04	11.02	0.001	0.70	0.497	2.49	0.085
biceps skinfold (mm)	8.02	3.80	7.05	3.76	6.37	3.34	7.73	2.88	7.79	2.84	8.39	3.21	2.15	0.145	2.79	0.063	11.62	0.000
triceps skinfold (mm)	12.73	3.50	11.90	3.69	10.86	3.52	12.13	3.36	12.16	2.79	14.16	3.68	3.41	0.067	1.64	0.195	22.33	0.000
subscapular skinfold (mm)	9.58	4.04	8.71	3.78	8.61	3.77	9.29	3.63	9.59	3.32	11.02	3.88	2.23	0.138	3.64	0.027	11.55	0.000
supraspinal skinfold (mm)	10.41	5.58	9.32	5.18	9.77	5.63	10.32	3.80	10.41	3.58	12.82	5.15	2.65	0.106	6.76	0.001	5.99	0.003
suprailiac skinfold (mm)	11.88	5.20	11.37	5.87	11.60	5.89	12.22	4.67	13.36	4.38	15.74	5.23	6.50	0.012	9.84	0.000	9.03	0.000
medial calf skinfold (mm)	11.70	4.15	11.83	4.19	10.69	4.20	11.96	3.55	12.30	3.45	13.78	3.63	4.49	0.036	0.85	0.431	9.94	0.000
lateral calf skinfold (mm)	12.33	3.45	12.50	3.89	12.05	3.70	12.26	3.17	12.49	3.29	15.14	4.30	3.38	0.068	9.56	0.000	12.96	0.000
thigh skinfold (mm)	16.63	4.61	15.54	4.68	14.59	4.93	17.66	4.50	17.76	4.63	20.58	5.39	16.70	0.000	2.55	0.080	15.65	0.000
sum of skinfold (mm)	93.25	29.36	88.53	31.38	83.93	30.12	93.57	23.71	95.86	23.38	111.63	28.56	5.84	0.017	5.46	0.005	22.52	0.000
%F	20.93	5.77	19.48	5.79	17.21	5.71	19.55	4.39	19.89	4.04	22.19	4.04	2.42	0.122	1.59	0.206	41.53	0.000
FM (kg)	9.49	3.81	9.88	4.17	9.74	4.26	8.83	2.91	9.95	3.06	11.99	3.26	0.52	0.472	38.35	0.000	24.81	0.000
FFM (kg)	34.64	5.38	39.38	5.90	45.32	7.03	35.48	4.97	39.18	4.59	41.38	4.49	1.87	0.174	440.94	0.000	41.47	0.000
club sport amount (h/week)	3.74	1.52	4.53	2.23	4.85	1.98	3.53	2.45	4.22	2.72	4.06	2.53	1.76	0.187	11.75	0.000	0.56	0.571

*BMI* Body Mass Index, *WHR* Waist-to-Hip Ratio, *WthR* Waist-to-Height Ratio, *%F* percentage of fat mass, *FM* Fat Mass, *FFM* Fat Free Mass, *Meas* measurement

Greater are the differences connected with the variation of the measurement over time. Except for cormic index, WHtR, three of skinfold thickness and %F, all the other characters presented a significant variation with age.

Analysing these variations in more detail, considering also the post-hoc, we can observe some different trend in the two sexes. In males, from the 1st to the 3rd class, height increment was 13.43 ± 4.24 cm, sitting height increment 6.21 ± 3.48 cm, leg length increment 7.23 ± 2.45 cm and weight increment 11.03 ± 4.15 kg.

The cormic index, which indicates the percentage of the sitting height on the total height, showed a slight tendency to decrease, with significant difference between the 1st and the 3rd class (*p* = 0.047). BMI presented a slight increase. On average, WHtR remained stable over the 3 years: a small percentage of boys presented WHtR values higher than the 0.5 cut-off (7.69% in the 1st class; 9.52% in the 2nd and 3rd classes). WHR showed a significant decrease with age.

In general, skinfold thicknesses remained quite stable over the 3 years, with a general tendency to decrease. However, the decrease observed for biceps, triceps and thigh skinfolds was significant (*p* < 0.05).

Fat Percentage significantly decreased with age, particularly between the 1st and the 3rd class (*p* < 0.001), while FFM significantly increased (p < 0.001).

Ninety-two percent of males practiced club sports. This percentage was constant in the first 2 years, with a slight decrease (90%) in the 3rd class. Weekly amount of club sports increased with age, with significant difference between the 1st and 3rd class.

In females, from the 1st to the 3rd class, height increment was 7.32 ± 3.51 cm, sitting height increment 4.40 ± 2.55 cm, leg length increment 2.92 ± 2.45 cm and weight increment 8.88 ± 4.15 kg. The cormic index did not show any significant variation with age, even though a tendency to increase was observed. BMI showed a significant increase between all the age classes (*p* < 0.001). Waist and hip circumferences showed a significant increase with age (*p* < 0.05), so that WHR showed a slight decrease with age, but not significant. WHtR did not show any significant differences during the 3 years, and also the subjects with values above the cut off remained stable over the 3 years (9.52%).

Almost all skinfold thicknesses showed a significant increase (*p* < 0.001), except for the biceps, subscapular and medial calf. This influenced body composition parameters, which significantly increased with age (p < 0.001). In particular, %F increased from 19.55% in the 1st class to 22.19% in the 3rd class.

Sports were practiced by 89.9% of the girls in the first class, passing to 87.1% in the second class and to 90% in the 3^rd^class. Weekly amount of club sports slightly increased during the 3 years, but not significantly.

### Weight status

Considering the prevalence of weight status in males (Table 2), no significant differences were observed among the 3 years. Normal weight prevailed in all the considered classes, showing an increasing trend with age. A small percentage of obese boys were observed in the 1st class, but this category disappeared in the following years, and a reduction of overweight subjects was observed.

**Table 2:** Prevalence of weight status (according Cole cut-off) in the different measurements

	I measurement (%)	II measurement (%)	III measurement (%)	p
***Males***				0.234
normal weight	68.75	76.19	77.78	
overweight	26.56	23.81	22.22	
obese	4.69	0.00	0.00	
***Females***				0.899
underweight	1.47	1.47	0.00	
normal weight	82.35	85.71	82.86	
overweight	14.71	12.83	17.14	
obese	1.47	0.00	0.00	

Similarly, in females the weight status prevalence did not significantly differ among the 3 years and normal weight subjects prevailed (Table 2). Contrarily to males, in the 1st and 2nd classes a small percentage of underweight girls were observed. Even if the differences were not significant, a slight increase in the prevalence of overweight in the 3rd class was observed.

### Maturity status

According to equation of Mirwald et al. [45] the mean age at PHV of the male sample was 14.00 years (SD = 0.62). The majority of the boys were average maturers (91.9%), only 3.2% were early maturers, and 4.8% late maturers. In the 3rd class about 93% of the subjects were approaching the PHV (distance from PHV = ± 0.5 years).

With reference to females, data regarding age at menarche showed that in the 1st class, 29.0% of the sample had reached it; this percentage increased to 62.9% in the 2^nd^class and to 74.3% in the 3rd class. The mean age at menarche was 11.97 (SD = 0.94 years).

### Body image perception

Body image perception showed significant differences between sexes (Table 3). The difference in the choice of perceived current figures was significant, since females have chosen thinner silhouettes than males. Body image dissatisfaction (FID) is greater in females. Males presented a greater misperception of their weight status, showing a greater underestimation than females.

**Table 3:** Body image perception by sex and repeated measures according to non-parametric ANOVA

	Males						Females						ANOVA					
	I measurement		II measurement		III measurement		I measurement		II measurement		III measurement		Sex difference		Meas. differences		Sex*Meas.	
Variables	Mean	SD	Mean	SD	Mean	SD	Mean	SD	Mean	SD	Mean	SD	F	p	F	p	F	p
perceived current figure	4.19	1.58	4.08	1.41	3.81	1.37	4.59	1.49	4.56	1.39	4.79	1.43	7.82	0.005	0.68	0.500	2.69	0.069
ideal figure	3.98	1.34	3.86	1.12	3.76	1.09	4.12	1.18	4.01	1.04	4.24	1.10	2.42	0.152	0.65	0.519	1.26	0.284
FID (score)	0.27	1.45	0.22	0.96	0.05	0.79	0.54	1.26	0.54	0.79	0.54	0.88	7.69	0.006	1.26	0.281	0.81	0.432
FAI (score)	−0.49	0.71	−0.44	0.64	−0.54	0.64	−0.09	0.78	−0.04	0.62	−0.04	0.67	25.13	0.000	0.29	0.731	0.38	0.667

*FID* Feel minus Ideal Discrepancy, *FAI* Feel weight status minus Actual weight status Inconsistency, *Meas* measurement

Body image parameters did not show any significant variations with age (Table 3). In all 3 years, on average, males chose normal weight silhouettes both for the perceived current figure and the ideal figure. As a consequence, the FID score indicated no dissatisfaction with their body, especially in the 3rd class. As regards the misperception of their weight status, FAI score indicated no variations with age; a tendency to underestimate the weight status was observed in all the three considered years.

On average, the perceived current figure chosen by girls (Table 3) belonged to the normal weight category, but it was in the upper extremity (nearly overweight). The mean ideal figure chosen also falls into the normal weight category, but presented lower values than the perceived current figure, as testified by positive mean values of FID, indicating a desire to be thinner. FAI score approximated zero, indicating no inconsistency in the perception of weight status, even though with a slight tendency to underestimation, testified by negative mean values.

### Predictors of FID and FAI

We performed different multiple linear regression models to evaluate the impact of age, sex and BMI on body image dissatisfaction (assessed by FID score) and on inconsistency of weight status (assessed by FAI score) in the adolescents during their 3 years of middle school (1st class, 2nd class, and 3rd class). The results of these predictive models are presented in Table 4.

**Table 4:** Predictors of FID (Feel minus Ideal Discrepancy) and FAI (Feel weight status minus Actual weight status Inconsistency): results of multiple regression analysis

Variables	I measurement			II measurement			III measurement		
FID	β	t	p	β	t	p	β	t	p
Age	−0.118	−1.488	0.139	−0.124	−1.827	0.070	−0.022	−0.373	0.710
BMI	0.402	5.056	0.000	0.596	8.821	0.000	0.675	11.176	0.000
Sex (female)	0.164	2.057	0.042	0.203	3.001	0.003	0.226	3.735	0.000
*R*^*2*^	0.183			0.407			0.533		
*Adjusted R*^*2*^	0.164			0.393			0.523		
*p*	0.000			0.000			0.000		
**FAI**	**β**	**t**	**p**	**β**	**t**	**p**	**β**	**t**	**p**
Age	0.024	0.285	0.776	−0.027	−0.343	0.732	0.109	1.403	0.163
BMI	0.054	0.642	0.522	0.307	3.885	0.000	0.289	3.711	0.000
Sex (female)	0.269	3.164	0.002	0.316	3.982	0.000	0.322	4.111	0.000
*R*^*2*^	0.074			0.189				0.222	
*Adjusted R*^*2*^	0.053			0.170				0.203	
*p*	0.018			0.000				0.000	

*β* regression coefficient, *BMI* Body Mass Index

As regards FID, both sex and BMI are significantly associated with FID and both are positive predictors. Body image dissatisfaction increases as BMI increases and being a female increases the dissatisfaction. The models explain 16% of the variance in the 1st class, 39% in the 2nd class and 52% in the 3rd class; all models have highly significant R^2^.

Being females is associated with a higher overestimation of one’s own weight status, as shown by the significant and positive association between sex and FAI (Table 4). In the transition to the 2nd class and 3rd class, the adolescents showed an increase of overestimation as their BMI increased. The models explain 5% of the variance in the 1st class, 17% in the 2nd class and 20% in the 3rd class; all models have highly significant R^2^.

## Discussion

### Trends in body composition and weight status

The present study considered the trends in body composition and in the prevalence of overweight and obesity, assessed using the Cole cut-off values [37, 38], in Italian adolescents followed longitudinally over the 3 years of middle school. In addition, another aim was to determine whether body composition and weight status during adolescence were associated with the risk of developing body image disturbance. To our knowledge, this study is the first to evaluate the longitudinal association between these parameters in young Italian adolescents.

As regards the first aim, the longitudinal variations in body composition of the subjects of the two sexes are connected with the phase of maturation they were approaching.

The male sample during the 3 years was approaching the growth spurt, and in the last year of measurement about 93% of the boys were near the time of PHV. This is reflected in the great height increment and in the prevalence of leg length growth in comparison with trunk growth. Differential timings of growth of height, sitting height and leg length are in fact known, since with growth a change in relationship between leg length and sitting height is observed. The growth of leg length precedes the PHV, while sitting height growth occurs after PHV [32]. The trend in parameters connected with body composition confirmed this specific stage of growth. During the 3 years, the sum of skinfolds showed a tendency to decrease in males, and, as a consequence, a significant decrease of %F with age was observed, accompanied by a significant increase of FFM were observed. These data are in accordance with the body composition and fat distribution changes that occur during male adolescence [32].

The percentage of mature girls increased with age, changing from 29.0% in the 1^st^class to 74.3% in the 3rd class. The mean age at menarche was 11.97 (SD = 0.94 years). A significant increase with age was observed for all the considered parameters, with height, weight, waist and hip circumferences showing significant differences among all the considered age classes. The height increment was lower than that observed in males, while the sitting height increment was higher than that of leg length: this reflects the different phase of maturation of the two sexes, with girls ahead compared to males.

Also connected with sexual dimorphism is the trend of parameters connected to body composition: skinfold thicknesses generally showed a significant increase in girls during the 3 years; it follows that the %F significantly increased from 19.55% in the 1st class to 22.19% in the 3rd class.

In males, BMI remained quite stable during the three school years. The percentage of normal weight males was lower than the national data [51], which reported an incidence of 75.9% in 11-years-old boys and 78.4% in 13-years-old boys, but it tended to approach these values with age. Percentages of overweight boys in the present sample were higher than the national data, but a tendency to decrease with age was observed. Obesity is slightly higher in the 1st class, but disappeared in the following years. Contrarily to the national data, there were no underweight subjects in the present sample. In girls, BMI showed a significant increase. Even if the weight status prevalence did not significantly differ among the 3 years and normal weight subjects prevailed, a reduction in underweight prevalence and a slight increase in the prevalence of overweight with age were observed. The percentage of normal weight girls was comparable to the national data [51], but the percentage of overweight girls was higher, and obese and underweight subjects were lower. Regarding sex differences in weight status, females showed a higher incidence of normal weight, and a lower incidence of overweight and obesity.

The latest data [52] from the WHO Childhood Obesity Surveillance Initiative (COSI) reveal that in countries like Italy, Portugal, Spain and Greece, there has been an important decrease of obesity, which is attributable to a very significant effort that these countries have made in recent years in the management and prevention of childhood obesity [53], although rates are still high. Despite the fact that the incidence of overweight in the present sample was high, the percentage of subjects engaged in sports practice (about 90% in both sexes) suggests that the issue of prevention was taken into account. Low levels of PA in adolescents is a critical cause for the obesity epidemic in developed countries [53, 54], and obese adolescents are generally less physically active (PA) than normal-weight adolescents [55]. As a consequence, increasing physical activity is probably one of the best strategies to reduce the prevalence of overweight and obesity in youth [56]. In addition, fat distribution may have more significant implications for health than the total amount of body fat. In the present study only a small percentage of subjects of both sexes presented a WHtR higher than cut-off. Visceral fatness or the accumulation of intra-abdominal adipose tissue increases cardiovascular risk in children, and it has been shown that that the most active individuals have the lowest visceral fat after adjusting for total body fat. This suggests that PA may elicit a proportionally larger decrease in fat stored in the intra-abdominal cavity than in other regions in people with visceral fatness [57, 58]. Cross-sectional data also suggest that PA could promote a reduction of visceral fat and trunk fat in pre-pubertal and pubertal children [59, 60]. In this longitudinal study, sports practice is high and regular, and once again this can be traced back to the preventive campaigns that the region in which the study took place [61] has put in place to encourage the spread of a healthy lifestyle among children and adolescents.

### Association between anthropometric change and body image perception

The second aim was to assess whether the change in body composition parameters and weight status influence body image perception. In both sexes, body image perception did not show any significant variations with age and both the perceived current figure and the ideal figures were chosen fall into the category of average in normal weight silhouettes. Nevertheless, females tend to choose a thinner ideal figure than the perceived current figure, as also testified by the FID. The average FAI index of both sexes is close to 0, implying a correct perception of their own weight status.

The data of the present study strengthen the notion that body image dissatisfaction and perception are mostly influenced by moderating factors such as sex and BMI, which should be considered in studies regarding youth. The results of the multiple linear regression analysis showed that BMI is a common denominator for both sexes, and as it increases also body dissatisfaction increases. With the increase of BMI, in the adolescents of the 2nd class and 3rd class, increases the overestimation of their weight status. In addition, girls are more vulnerable than males for both dissatisfaction. In girls, fat mass increases more rapidly than in boys during adolescence. Important variations in body composition, which are influenced by age and sex, and which include BMI, subcutaneous fat and relative fat distribution [31, 62–64] can be sources of dissatisfaction in body image perception.

The results of the present study are in accordance with those of other Authors [65–67], which showed that increased BMI is a key point in influencing body image perception. It is associated with lower self-perceptions of social acceptance and physical appearance, and subjects who demonstrate low perceptions of self-concept are also generally less willing to participate in activities with peers [68–70].

### Strength and limits

The strength of this study is the three-year longitudinal design in which anthropometric parameters of the adolescents were directly measured by an expert anthropometrist, and not self-reported.

A limitation of this study could be connected to the use of standardized body silhouettes instead of computerized assessments, which allow to assess separate characteristics of body image, such as overall size or shape. This technological method presents a good flexibility to visually represent how users perceive themselves, compared to fixed silhouettes. In addition, it allows detailed information to be gathered regarding the assessments of individual body areas [71, 72]. Even if the silhouettes used in the present study are widely utilized in the literature [73, 74] they do not allow the assessments of muscularity, an important aspect considered in studies on older subjects [75, 76].

## Conclusion

Adolescence is a complex transition stage, during which, in relation to maturation, significant changes in body composition occur. The adolescents of the present study are in the phase where these changes take place, and where body fat percentage increases in females and decreases in males. Even if their body image perception did not seem to change with age, associations were found between body image perception and BMI and sex. Dissatisfaction and overestimation of one’s own weight status increase as BMI increases, and in females to a greater extent than in males. The results of the present study do not confirm the initial hypothesis that adolescence in itself leads to an increase in body image misperception, but confirms the greater dissatisfaction of females and of overweight/obese subjects. Monitoring body image perception in young adolescents, especially in females and in subjects with a high BMI, should be a priority in order to prevent nutritional disorders.

## Data Availability

The datasets used during the current study are available from the corresponding author on reasonable request.

## Abbreviations

FAI: Feel weight status minus Actual weight status Inconsistency
WHO: World Health Organization
ED: Eating disorders
BMI: Body mass index
WHR: Waist/Hip ratio
WHtR: Waist/height ratio
%F: Percentage of fat mass
FM: Fat mass
FFM: Fat free mass
PHV: Peak height velocity
FID: Feel Ideal Discrepancy
HBSC: Health Behaviour in School-aged Children
PA: Physical Activity
COSI: Childhood Obesity Surveillance Initiative
